# Hepatitis E Virus Infections in Blood Donors, France

**DOI:** 10.3201/eid2011.140516

**Published:** 2014-11

**Authors:** Pierre Gallian, Sébastien Lhomme, Yves Piquet, Karine Sauné, Florence Abravanel, Azzedine Assal, Pierre Tiberghien, Jacques Izopet

**Affiliations:** École des Hautes Études en Santé Publique, Marseille, France (P. Gallian); Etablissement Français du Sang Alpes Méditerranée, Marseille (P. Gallian);; **Institut National de la Santé et de la Recherche Médicale Unite 1043,** Toulouse, France (S. Lhomme, K. Sauné, F. Abravanel, J. Izopet);; Université Toulouse III Paul-Sabatier, Toulouse (S. Lhomme, K. Sauné, F. Abravanel, J. Izopet);; Institut Fédératif de Biologie, Toulouse (S. Lhomme, K. Sauné, F. Abravanel, J. Izopet);; Etablissement Français du Sang Aquitaine-Limousin, Bordeaux, France (Y. Piquet, A. Assal);; Etablissement Français du Sang, La Plaine Saint-Denis, France (P. Tiberghien);; Université de Franche-Comté, Besançon, France (P. Tiberghien)

**Keywords:** hepatitis E, hepatitis E virus, HEV, viruses, HEV RNA, viremia, blood donors, solvent–detergent, plasma, blood transfusions, France

## Abstract

We screened plasma samples (minipools of 96 samples, corresponding to 53,234 blood donations) from France that had been processed with solvent–detergent for hepatitis E virus RNA. The detection rate was 1 HEV-positive sample/2,218 blood donations. Most samples (22/24) from viremic donors were negative for IgG and IgM against HEV.

Hepatitis E virus (HEV), family *Hepeviridae*, genus *Hepevirus*, is a small nonenveloped RNA virus that has an icosahedral capsid and is transmitted by the fecal–oral route (*1*). Transfusion-transmitted HEV infections have been documented in several countries in Europe and Asia (*2**–**4*). HEV has also been detected in human blood products (*5**–**12*). Despite considerable geographic variation in HEV seroprevalence in Europe, southern France seems to be an area to which this virus is hyperendemic (*13*).

We conducted a prospective study in France of minipools of plasma from blood donations that were processed with solvent–detergent and used standardized molecular assays for detection of HEV RNA. Data obtained were used to estimate the detection rate of HEV infections in blood donors in France and the risk for HEV transmission by blood transfusion.

## The Study

**Figure 1:**
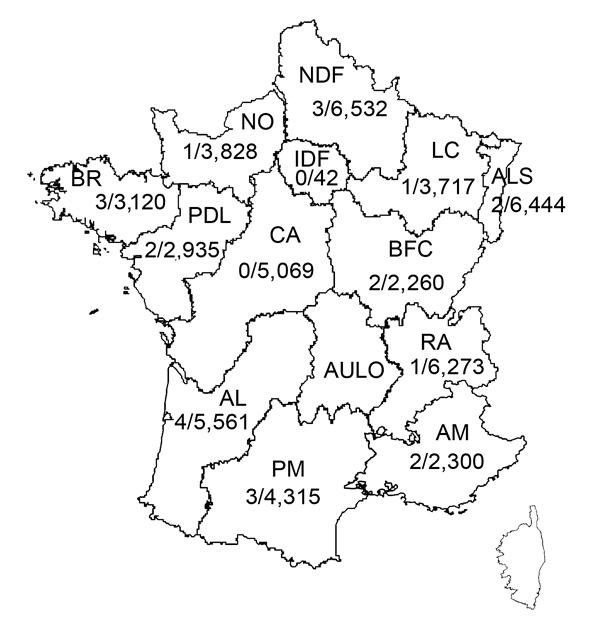
Prevalence (no. samples positive/no. tested) of hepatitis E virus RNA in plasma collected during November 27, 2012–December 1, 2013, at regional establishments of the French Blood Agency, France. Southern France: AL, Aquitaine-Limousin; PM, Pyrénées-Méditerranée; AM, Alpes-Méditerranée (including Corsica). Northern France: NDF, Nord de France; NO, Normandie; IDF, Ile de France; LC, Lorraine-Champagne; ALS, Alsace; BR, Bretagne; PDL, Pays de Loire; CA, Centre Atlantique; BFC, Bourgogne-France Comté; AULO, Auvergne-Loire; RA, Rhône-Alphes.

Since November 27, 2012, systematic nucleic acid amplification screening for HEV has been used on blood donations (pools of 96 samples) in France for a 70-L plasma pool that was processed with solvent–detergent. A mixture of 96 plasma samples (50 µL from each sample) was prepared by using a distributor (Tecan, Männedorf, Switzerland) to give a final volume of 4.8 mL. Simultaneously, a 96-well archive microplate was used for serologic testing and to identify individual HEV RNA–positive samples. Plasma samples were collected at 13/14 regional blood transfusion establishments of the French Blood Service in continental France (Figure 1).

HEV RNA was extracted by using the Nuclisens Easy MAG Instrument (bioMérieux, Marcy-l’Etoile, France). Amplification was performed by using the single-round RealStar Reverse Transcription PCR (Altona Diagnostics, Courtaboeuf, France), which is specific for the open reading frame 2 (ORF2)–ORF3 overlapping region (*12*); the 95% detection limit was 23 IU/mL. HEV RNA quantification was performed as described (*14*); the limit of quantification was 60 IU/mL. HEV genotyping was performed by sequencing a 305-nt fragment within the ORF2 region and performing phylogenetic analysis.

IgG and IgM against HEV were detected by using an enzyme immunoassay (Wantai Biologic Pharmacy Enterprise, Beijing, China) according to the manufacturer’s instructions (*13*). World Health Organization reference material for IgG against HEV was used to determine concentrations of IgG against HEV (*15*). The limit of detection was 0.25 World Health Organization U/mL.

We screened 558 pools (53,234 samples) for HEV RNA. Of these pools, 22 (3.94%) showed positive results (Table 1). We detected 1 HEV RNA–positive sample in each of 20 pools and 2 HEV RNA–positive samples in the remaining 2 pools. The estimated detection rate of HEV RNA in plasma donations was 0.045% (95% CI 0.043%–0.047%).

**Table 1:** Characteristics of 22 HEV RNA–positive plasma pools, France, November 27, 2012–December 1, 2013*

Pool	HEV RNA, IU/mL	IgG titer against HEV, U/mL	IgM against HEV
P1	<60	+, <0.30	–
P2	578	+, 0.80	–
P3	3,978	+, 0.30	–
P4	415	+, 2.70	–
P5	200	+, 1.00	–
P6	<60	+, 1.10	–
P7	1,458	+, 1.60	–
P8	<60	+, 0.32	–
P9	<60	NT	NT
P10	5,374	+, NT	–
P11	29,796	+, 0.56	–
P12	<60	+, 1.93	–
P13	<60	+, 0.42	–
P14	<60	+, NT	–
P15	2,948	+, 0.49	–
P16	171	+, 1.44	–
P17	2,137	+, 0.44	–
P18	<60	–	–
P19	<60	+, 0.84	IND
P20	3,705	+, 0.26	–
P21	<60	+, 0.81	–
P22	<60	+, 2.61	–

*HEV, hepatitis E virus; +, positive; –, negative; NT, not tested because of insufficient material; IND, indeterminate.

**Figure 2:**
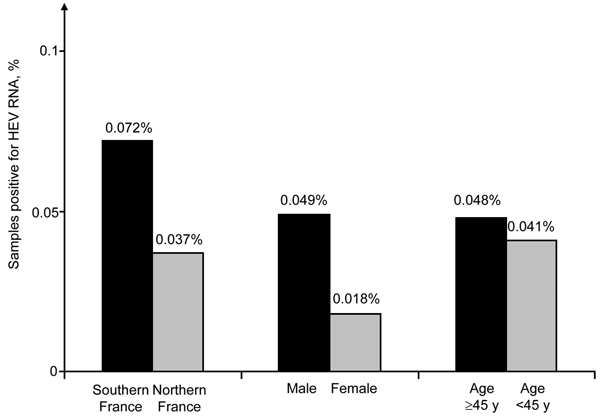
Frequency of blood donations positive for hepatitis E virus (HEV) RNA, by location and donor age and sex, France, November 27, 2012–December 1, 2013. Comparisons between groups by using χ^2^ and Fisher exact tests showed no significant differences.

The geographic distribution of HEV-positive samples included all but 2 blood transfusion establishments (in Centre Atlantique and Ile de France) (Figure 1). The frequency of HEV RNA–positive samples was 0.072% (95% CI 0.067%–0.077%) in 3 blood transfusion establishments in southern France and 0.037% (95% CI 0.035%–0.037%,) in establishments in northern France (odds ratio [OR] 1.98, 95% CI 0.86–4.53, p = 0.14) (Figure 2). The frequency of HEV RNA–positive samples was 0.049% (95% CI 0.047%–0.051%) in men and 0.018% (95% CI 0.014%–0.022%) in women (OR 2.7, 95% CI 0.4–111.1, p = 0.51). The frequency of HEV RNA–positive samples was 0.048% (95% CI 0.046%–0.052%) for persons ≥45 years of age and 0.041% (95% CI 0.040%–0.042%) for persons <45 years of age (OR 1.2, 95% CI 0.5–2.9, p = 0.83).

HEV RNA concentrations in 11 pools were below the limit of quantification, and the plasma pool with the highest virus load contained 29,796 IU/mL (Table 1). The range of virus load in individual plasma samples was 468 IU/mL–5,155 800 IU/mL. All strains that were available for sequence analysis were assigned to HEV genotype 3 (Technical Appendix). A total of 59% of the strains were subgenotype 3f and 36% were subgenotype 3c (Table 2).

**Table 2:** Characteristics of 24 blood donors with HEV RNA–positive archived blood samples, France, November 27, 2912–December 1, 2013*

Donor/age, y/sex	HEV RNA, IU/mL	Genotype	IgG against HEV	IgM against HEV
1/32/M	1,903	3f	–	–
2/37/M	128,700	3f	–	–
3/60/M	1,404,100	3f	–	–
4/52/F	179,400	3c	–	–
5/55/M	102,960	3c	–	–
6/59/M	19,344	3f	+	+
7/41/M	1,333	3f	–	–
8/61/M	391,560	3c	–	–
9/28/M	2,293	3f	–	–
10/40/M	8,502	3	–	–
11/57/M	803,400	3f	–	–
12/37/M	5,155,800	3c	–	–
13/37/M	1,981	3f	–	–
14/58/M	6,208	3c	–	–
15/53/M	13,728	3c	–	–
16/59/M	111,696	3f	–	–
17/44/M	61,932	3c	–	–
18/54/M	258,180	3c	–	–
19/34/M	4,852	3f	–	–
20/41/M	3,994	3f	–	–
21/48/M	468	NT	+	+
22/55/M	306,540	3f	–	–
23/25/M	4,118	3f	–	–
24/57/M	2,355	NT	–	–

*HEV, hepatitis E virus; –, negative; +, positive; NT, not tested because of insufficient material.

Of the 183 pools tested for IgG against HEV, 175 had positive results and 8 eight had indeterminate results. Of these 175 IgG-positive pools, 173 were negative for IgM against HEV; the remaining 2 pools had indeterminate results. The range of IgG titers against HEV was 0.3 U/mL–10.6 U/mL.

All HEV RNA–positive pools were negative for IgM against HEV and positive for IgG against HEV, except for 1 pool that was negative for IgG against HEV (Table 1). IgG titers against HEV in viremic pools ranged from 0.3 U/mL to 2.7 U/mL. The prevalence of IgG against HEV for 861 blood donors (whose samples were included in the first 9 HEV RNA–positive pools) was 23.6%. IgG-positive pools included 13.5%–30.2% of plasma collected from blood donors who were positive for IgG against HEV. Pools negative for IgG included 10% of IgG-positive donations. Most (22/24, 91.7%) viremic blood donors were negative for IgM and IgG against HEV at the time of their donation, which indicated recent infection. Two donors (D6 and D21) were positive for IgG and IgM against HEV (Table 2).

## Conclusions

We conducted a prospective study with a sensitive method to screen plasma pools for HEV RNA and found that the frequency of HEV infections in donated blood in France was relatively high (1/2,218). This is 1 of the highest frequencies reported for Europe. Frequencies in Europe were 1/14,520 in Scotland (*7*), 1/7,040 in England (*10*), 1/4,525 and 1/1,240 in Germany (*5**,**12*) and 1/2,700 in the Netherlands (*11*).

Our study used plasma pools that contained 96 samples. Therefore, the true frequency of viremic samples could have been underestimated because of a dilution effect. The limit of detection of the PCR was low (<23 IU/mL). However, some pools could have been missed, although 5 positive individual donations having HEV RNA concentrations of 468 IU/mL–2,293 IU/mL were detected (Table 2).

The detection rate for viremic blood donations in southern France was 2-fold greater than that for the rest of France. This finding is consistent with a previously reported high seroprevalence (52%) of IgG against HEV in blood donors (*13*) and the high incidence (3.2%) of infection documented by molecular techniques in local transplantation patients (*15*). However, HEV-positive blood donations were detected in all but 2 of the regions of continental France tested. All strains were genotype 3 and the proportions of subgenotypes 3f and 3c strains was similar to those observed in HEV-infected pigs in France, which suggested a zoonotic origin of the 24 HEV infections as reported by Kamar et al. (*1*).

Infections were detected at an early stage because serologic markers were absent for >90% of the cases. Since November 2012, testing for HEV RNA has reduced the risk for HEV transmission in patients in France who are given plasma processed with solvent–detergent. This testing helps overcome the situation that this treatment does not inactivate HEV, a nonenveloped virus. In January 2015, nucleic acid amplification testing for HEV will begin in Europe for plasma processed with solvent–detergent.

We detected IgG against HEV in nearly all plasma pools, and IgG concentrations ranged from 0.3 U/mL to 10.6 U/mL. The potential of antibodies against HEV to neutralize virus infectivity if the unit is transfused is unknown, but a recent study indicated that IgG against HEV at a concentration of <10 U/mL cannot protect immunocompromised patients against reinfection (*15*).

Our findings should be useful for determining the best safety measures to prevent HEV transmission by blood transfusion. The implementation of nucleic acid amplification testing relies on full assessment of transfusion risk in exposed patients and the cost-effectiveness of different strategies.

Technical Appendix Phylogenetic analysis of hepatitis E virus RNA sequences by using the neighbor-joining method and a Kimura 2-parameter distance matrix based on a 305-nt fragment of open reading frame 2.
