# Supplementary material for: Hepatitis E Virus Infections in Blood Donors, France
Source: Emerg Infect Dis. 2014 Nov;20(11):1914–7. doi: 10.3201/eid2011.140516 (PMC4214305; doi:10.3201/eid2011.140516)
Supplement: Technical Appendix — Phylogenetic analysis of hepatitis E virus RNA sequences by using the neighbor-joining method and a Kimura 2-parameter distance matrix based on a 305-nt fragment of open reading frame 2. [file 14-0516-Techapp-s1.pdf]

Article DOI: http://dx.doi.org/eid2011.140516

# Hepatitis E Virus Infections in Blood Donors, France

## Technical Appendix

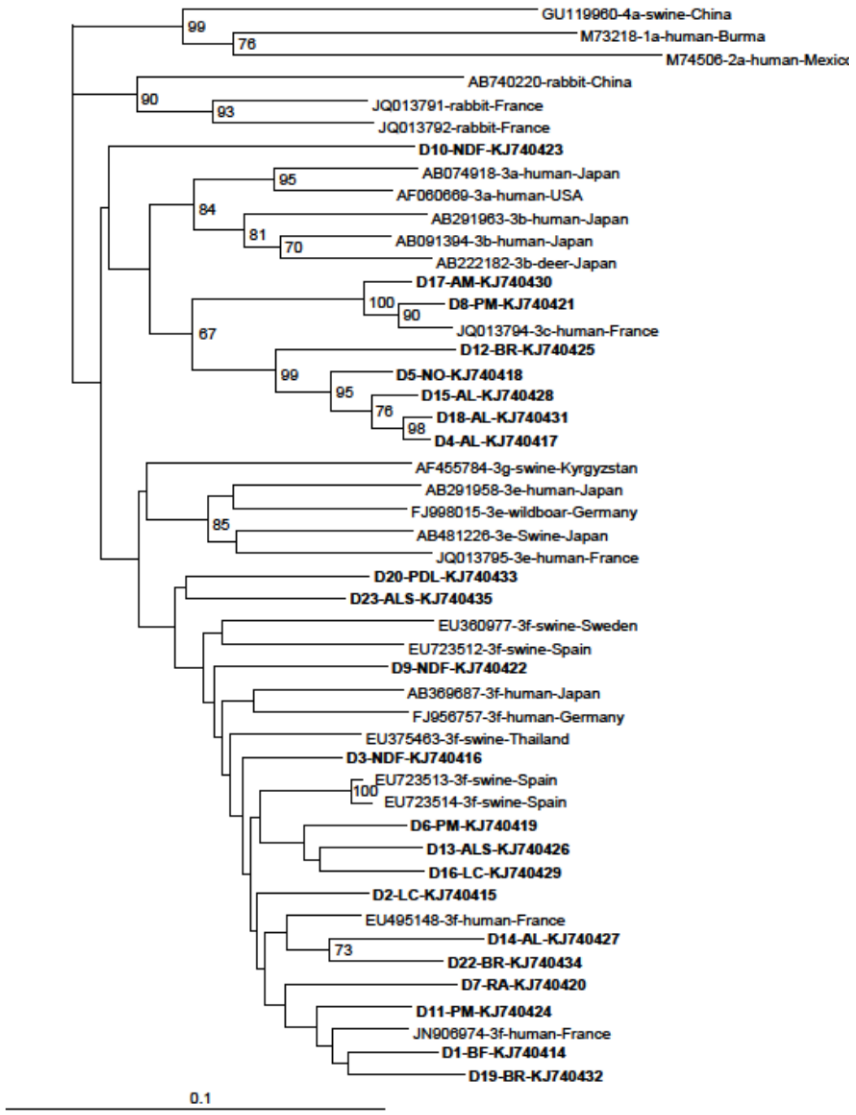


Technical Appendix Figure. Phylogenetic analysis of hepatitis E virus RNA sequences by using the neighbor-joining method and a Kimura 2-parameter distance matrix based on a 305-nt fragment of open reading frame 2. GenBank accession nos. for isolates from this study (in bold) are KJ740414–KJ740435. Values along the branches are bootstrap values, which are indicated as a percentage of the data obtained from 1,000 resamplings. Scale bar indicates nucleotide substitutions per site.
